# Metformin: Beyond Type 2 Diabetes Mellitus

**DOI:** 10.7759/cureus.71730

**Published:** 2024-10-17

**Authors:** Rahnuma Ahmad, Mainul Haque

**Affiliations:** 1 Department of Physiology, Medical College for Women and Hospital, Dhaka, BGD; 2 Department of Pharmacology and Therapeutics, National Defence University of Malaysia, Kuala Lumpur, MYS

**Keywords:** hepatic diseases and enzymes, inflammation and oxidative stress, insulin sensitivity, lifestyle, lipophagy, liver enzymes, metformin, nafld, novel treatment protective mechanism and healing, pharmacovigilance

## Abstract

Metformin was developed from an offshoot of Guanidine. It is known to be the first-line medication for type 2 diabetes mellitus, polycystic ovarian syndrome, and weight reduction. Metformin has also been shown to have effectiveness in the management of non-alcoholic fatty liver disease (NAFLD), liver cirrhosis, and various carcinomas like hepatocellular, colorectal, prostate, breast, urinary bladder, blood, melanoma, bone, skin, lung and so on. This narrative review focuses on the effect of metformin on non-alcoholic fatty liver disease, liver cirrhosis, and hepatocellular carcinoma. The search platforms for the topic were PubMed, Scopus, and Google search engine. Critical words for searching included 'Metformin,' AND 'Indications of Metformin,' AND 'Non-Alcoholic Fatty Liver Disease,' AND 'Metformin mechanism of action,' AND 'NAFLD management,' AND 'NAFLD and inflammation,' AND 'Metformin and insulin,' AND 'Metformin and inflammation,' AND 'Liver cirrhosis,' AND 'Hepatocellular carcinoma.' Lifestyle modification and the use of hypoglycemic agents can help improve liver conditions. Metformin has several mechanisms that enhance liver health, including reducing reactive oxygen species, nuclear factor kappa beta (NF-κB), liver enzymes, improving insulin sensitivity, and improving hepatic cell lipophagy. Long-term use of metformin may cause some adverse effects like lactic acidosis and gastrointestinal disturbance. Metformin long-term overdose may lead to a rise in hydrogen sulfide in liver cells, which calls for pharmacovigilance. Drug regulating authorities should provide approval for further research, and national and international guidelines need to be developed for liver diseases, perhaps with the inclusion of metformin as part of the management regime.

## Introduction and background

**Figure 1:**
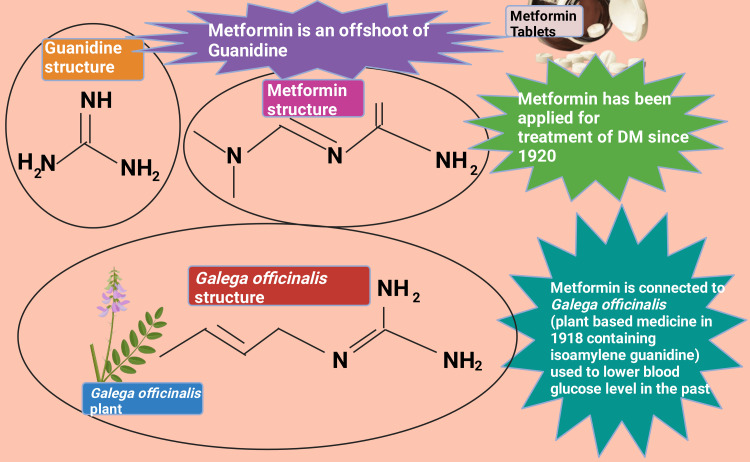
Metformin origin and history. DM: diabetes mellitus. This figure was drawn using the premium version of BioRender (https://biorender.com/), accessed on October 9, 2024, with the agreement license number PI27EIRU2R [10]. Image credit: Rahnuma Ahmad.

Metformin (dimethylbiguanide) was first introduced in France in 1957 by the French physician Jean Sterne (1909-1997) [1]. However, metformin was earliest portrayed in a scholarly peer-reviewed scientific journal by Emil Werner and James Bell in 1922 [2]. Metformin narration is connected to Galega officinalis (also known as Goat's rue, French lilac, Italian fitch, Spanish sainfoin, professor weed), a long-established plant-originated medicine in medieval Europe in 1918 [3-5]. Galega officinalis extract contained a considerable portion of isoamylene guanidine (galegine) and was demonstrated to lower blood glucose in 1918 [6-9]. Metformin, an offshoot of guanidine, was applied to treat type 2 diabetes mellitus (T2DM) from 1920 to 1930 (Figure 1) [1,5,6,10-12]. Nonetheless, Galega officinalis clinical utilization was ended due to adverse drug reaction (ADR) and the increased availability of insulin [1,13]. It was evidenced that metformin possesses antiviral potential [1,14-18]. This beneficial antiviral pharmacology was observed by scientists exploring anti-malarial medicine in the 1940s [1,19]. Nonetheless, metformin, from time to time, brings down blood glucose levels while treating influenza [1,14,20].

Diabetes mellitus (DM) is a persistent diverse metabolic disorder that has become a global epidemic and is principally caused by low synthesizing (availability) endogenous insulin from beta-cells of the pancreas and reduced sensitivity [21,22]. There are types of DM: type 1 (Insulin-dependent DM (IDDM)) and type 2 (non-insulin-dependent DM (NIDDM)) [23]. T2DM (NIDDM) is also acknowledged as adult-onset diabetes and comprises around 90-95% of all cases of DM [21,24,25]. T2DM is illustrated by two dominant insulin-associated incongruities: insulin resistance and β-cell dysfunction [26,27]. Globally, T2DM is considered the principal impelling force behind the death of 1.6 million individuals [28]. Largely, metformin is regarded as the first-line medication for T2DM [29]. Sharma et al. (2016) reported that 83.6% of British T2DM patients were prescribed metformin [30]. Pandya et al. (2023) reported that metformin occupies the bulk share (two-thirds) of the oral glucose-lowering medications in the USA. Additionally, metformin prescribed among T2DM cases receiving any rally consumed medication were 64%,66%,67%, 68%, and 68% in 2016, 2017, 2018, 2019, and 2020, respectively [31]. Overbeek et al. (2017) reported that metformin remains the most preferred blood glucose-lowering medication across all European countries, and utilization of this euglycemic agent has been observed to increase [32].

**Figure 2:**
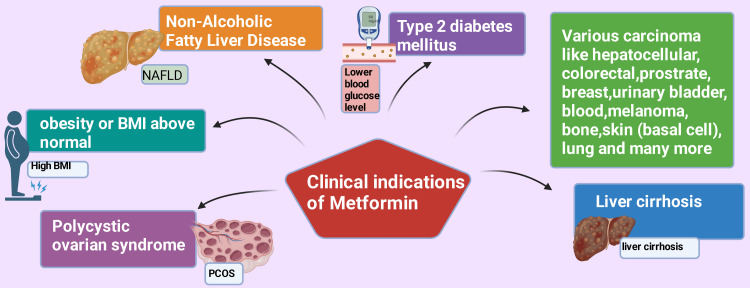
Clinical indications of metformin. BMI: body mass index. This figure was drawn using the premium version of BioRender (https://biorender.com/), accessed on October 2, 2024, with license number AF27DL49UX [10]. Image credit: Rahnuma Ahmad.

Naseri et al. (2022) reported that metformin is primarily prescribed for T2DM, PCOS, and weight reduction [33]. Various research studies are currently being conducted regarding metformin, and other reasonable clinical indications are transpiring that this medicine can be applied for purposes other than DM [34-38]. Those clinical indications include non-alcoholic fatty liver disease (NAFLD) [39], liver cirrhosis [40], various carcinoma, such as hepatocellular (HCC) [41,42], colorectal [43], prostate [44-46], breast [47,48], urinary bladder [49-51], blood [52,53], melanoma [54-56], bone [57-59], skin (basal cell) [60], lung [61,62], and many more (Figure 2). This narrative review paper will primarily concentrate on NAFLD, liver cirrhosis, and hepatocellular carcinoma.

## Review

Materials and methods

**Figure 3:**
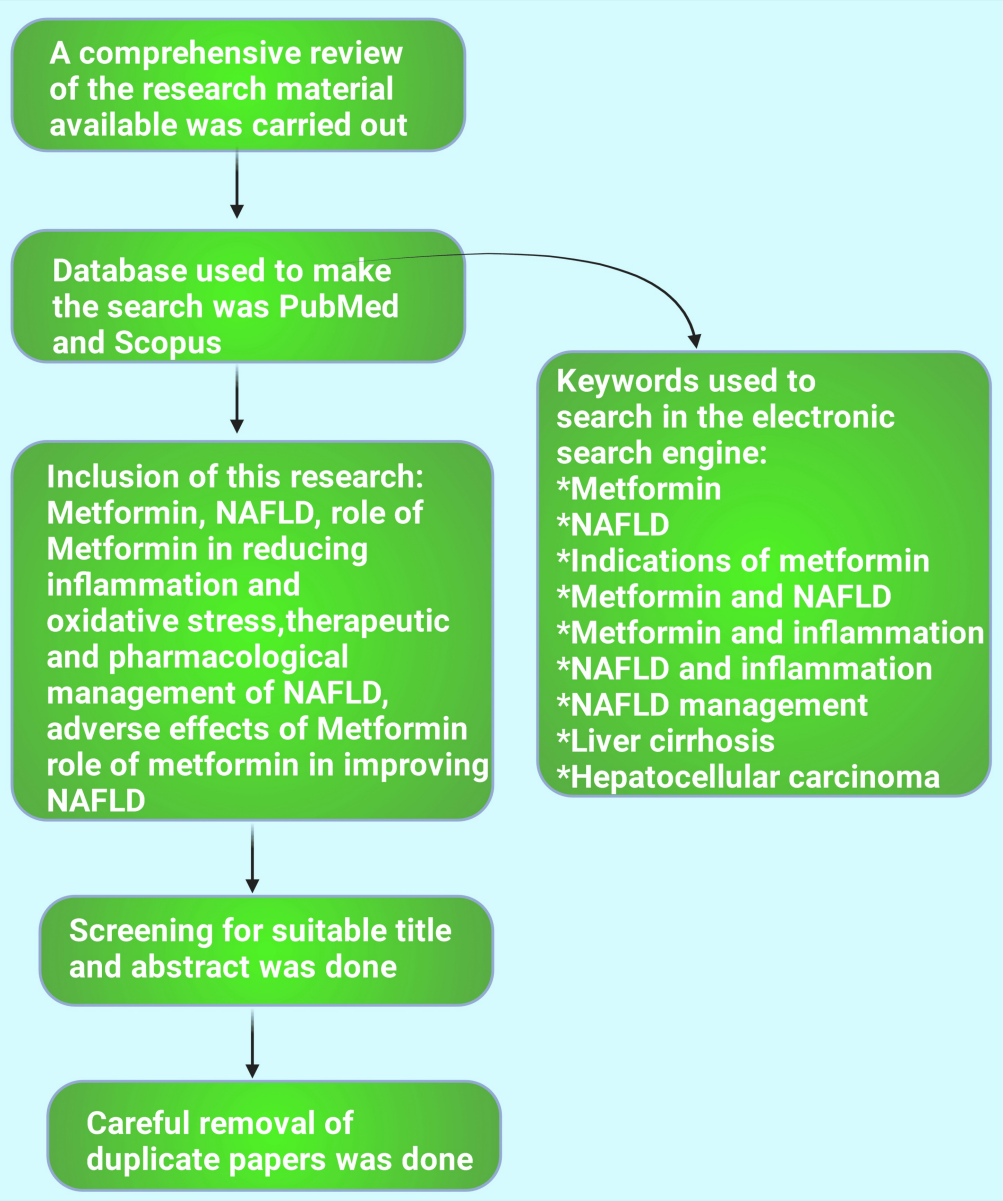
Flowchart depicting the materials and method section of the current study. NAFLD: Non-alcoholic fatty liver disease. This figure was drawn using the premium version of BioRender (https://biorender.com/), accessed on September 20, 2024 with the agreement license number DV27CQSXP4 [10]. Image credit: Rahnuma Ahmad.

This narrative review delves into the role of metformin in managing conditions like non-alcoholic fatty liver disease. Research has also been carried out on the literature available regarding the current epidemiology of NAFLD and the possible therapeutic and pharmacological management of NAFLD. The role of metformin in reducing oxidative stress and inflammation and its effect on improving NAFLD have also been highlighted. The information needed for this research was gathered between July 2024 and September 2024, employing the data offered by Scopus, PubMed, and Google Scholar. Keywords for the search were 'Metformin,' AND 'Indications of Metformin,' AND 'Non-Alcoholic Fatty Liver Disease,' AND 'Metformin mechanism of action,' 'NAFLD management,' AND 'NAFLD and inflammation,' AND 'Metformin and insulin,' AND 'Metformin and inflammation,' AND 'Liver cirrhosis,' AND 'Hepatocellular carcinoma' (Figure 3).

Review of the literature

Non-alcoholic Fatty Liver Disease

Global epidemiology of NAFLD: NAFLD is a comprehensive appellation for a range of disorders when fatty degeneration is detected through histopathological examination over 5% of hepatic cells and concurrently presence of metabolic syndrome precepting features (predominantly T2DM and obesity), disregard of excessive regular alcohol drinking or other long-lasting liver diseases [63-65]. Teng et al. (2023) reported that NAFLD is a prominent basis of hepatic disorders globally. It has been appraised that worldwide incidence among 1,000 populace 47 suffers from NAFLD and more seen among adults and males in comparison to pediatric community and females, respectively [66]. Riazi et al. recently published one systematic review and meta-analysis appraising that over 32% of adult people around the globe were stricken by NAFLD [67]. Another similar study by Younossi et al. 2023 revealed that over 30% of the population of our planet was suffering from NAFLD [68]. Multiple studies reported that in the past 30 years, the prevalence of NAFLD increased from 25 to 38% [68]. The maximum NAFLD frequency was in Latin America 44.37% (30.66%-59.00%), then the Middle East and North Africa (MENA) (36.53%, 28.63%-45.22%), South Asia (33.83%, 22.91%-46.79%), Southeast Asia (33.07%, 18.99%-51.03%), North America (31.20%, 25.86%-37.08%), East Asia (29.71%, 25.96%-33.76%), Asia Pacific 28.02% (24.69%-31.60%), and Western Europe 25.10% (20.55%-30.28%) [68]. Multiple studies reported that the worldwide occurrence of NAFLD was 38% [68-70]. It has been reported that the occurrence of NAFLD in the USA increased from 38 to 50% in the last three decennaries [70]. Ye et al. (2020) reported that in some nations, such as Malaysia and Pakistan, NAFLD was 25% or lower among non-obese subjects. Nonetheless, the prevalence was 50% or more in Mexico, Sweden, and Austria [71].

Asian epidemiology of non-alcoholic fatty liver disease: In Asia, for example, NAFLD-related health liability was detected in the uppermost (51.04%) and bottommost (22.28%) areas of Indonesia and Japan, respectively [72]. In India, the occurrence of NAFLD in both sexes was similar and generally pooled a commonness of 38.6% and 35.4% among adults and pediatric cases [73]. Various research groups reported that the Chinese mainland population had NAFLD ∼15% [74], 29.6% [75], 30% [76], 36.9% [77], and 44.39% [78]. However, another study conducted in Shanghai, China, reported that 5.07% of the pediatric population had ﻿fatty liver disease (FLD) [77]. The global prevalence of children and adolescents (below 18 years) NAFLD differs inter and intra-country. Among the pediatric obese population, it was 52.49% and 7.40% in non-obese pediatric cases. It has been estimated to reach up to 30.7% by 2040" [79]. The prevalence of primarily over ¼ of the Japanese population, men, was statistically (p<0.001) higher than women. It has been estimated that 39.3% and 44.8% of Japan's population will possibly be affected by NAFLD by 2030 and 2040, respectively [80]. A systematic review and meta-analysis were conducted among studies of the Kingdom of Saudi Arabia (KSA). Eight studies that included 4045 adult NAFLD cases were included. The pooled incidence of NAFLD among the study participants was 16.8% (11.1-22.5%). Additionally, 58% (45-70.9%) of these NAFLD were concurrently suffering from T2DM [81]. It has been estimated that NAFLD incidence in KSA will go beyond 30% by 2030 [82]. The prevalence rate of NAFLD in Indonesia is 51% [83]. In Malaysia, two studies published in 2013 and 2018 reported that NAFLD was 22.7% and 37.4%, respectively [84,85]. Proton-magnetic resonance spectroscopy and transient elastography are identified as exceedingly precise diagnostic devices to determine hepatic fatty degeneration in one most extensive population‐based analysis among the Asian population revealed that NAFLD is considerably increasing in this continent. In these studied populations, 80% and 5% had all five components without any features of metabolic syndrome (MetS) [86].

Therapeutic Intervention of NAFLD

Mayo Clinic of the United States of America recommended that medical intervention for NAFLD typically begins with reducing body weight. Consumption of a healthy nutritional diet, strictly avoiding energy-dense carbohydrate-containing food, and restraining amount of food and aerobic physical activity exercise. Reducing body weight and obesity often helps to minimize other potential health disorders that lead to NAFLD. Archetypally, it has been advised that lowering body weight by 10% or more has a beneficial impact on NAFLD [87]. It has been reported that more weight loss (10% or more) offers more benefits for NAFLD cases and possibly overthrows fatty liver hepatitis and even hepatic fibrosis [88]. ﻿Lifestyle intercessions constructed on modest to intense physical activity and a healthy eating plan and practice remain the principle of NAFLD non-pharmacological management [89-92]. It has advocated that "the Mediterranean diet is regarded as the diet of choice for the prevention/treatment of NAFLD and its complications, based on the available evidence" [93].

Dietary restriction and increased physical activity persist in the strategic remedial components to combat the worldwide health-related heavy impediment of hepatic fatty degeneration disorders [94-99]. Multiple studies reported that consuming low-energy-dense (strict avoidance carbohydrate) food, thereby limiting high-energy units, positively impacts MetS and minimizes the severity of NAFLD [100-103]. Various studies reported that sporadic energy-constraint food consumption (rigorous cutback of carbohydrate-rich foods) promotes ketogenesis and appears as the principal systematic feature of dietary interventions for managing NAFLD [104-110]. The ketogenic diet is an efficient intervention for the management of NAFLD. It is substantiated that liver "mitochondrial fluxes and redox state" are noticeably transformed throughout the ketogenic diet-persuaded improvement of NAFLD in humans [104].

Adequate aerobic physical exercise of modest intensity (150-240 minutes per week) single-handedly salvages abnormal preservation of lipids (fat) in the liver and viscera, avert fibrosis and cirrhosis, and diminish fatal outcomes [111-113]. Health-enriching physical exercise drops the possibility of the development of equally obese NAFLD and non-obese NAFLD among Asians. In contrast, the likelihood of developing NAFLD among slim individuals was considerably minimal, even if they were nominally doing physical exercise compared to inactive lean cases [114,115]. Skeletal muscle often demarcated an endocrine organ [116,117] discharges cytokines and myokines while contracting or functioning. These cytokines and myokines possess anti-inflammatory properties, especially among hepatic and adipose tissue [118].

**Figure 4:**
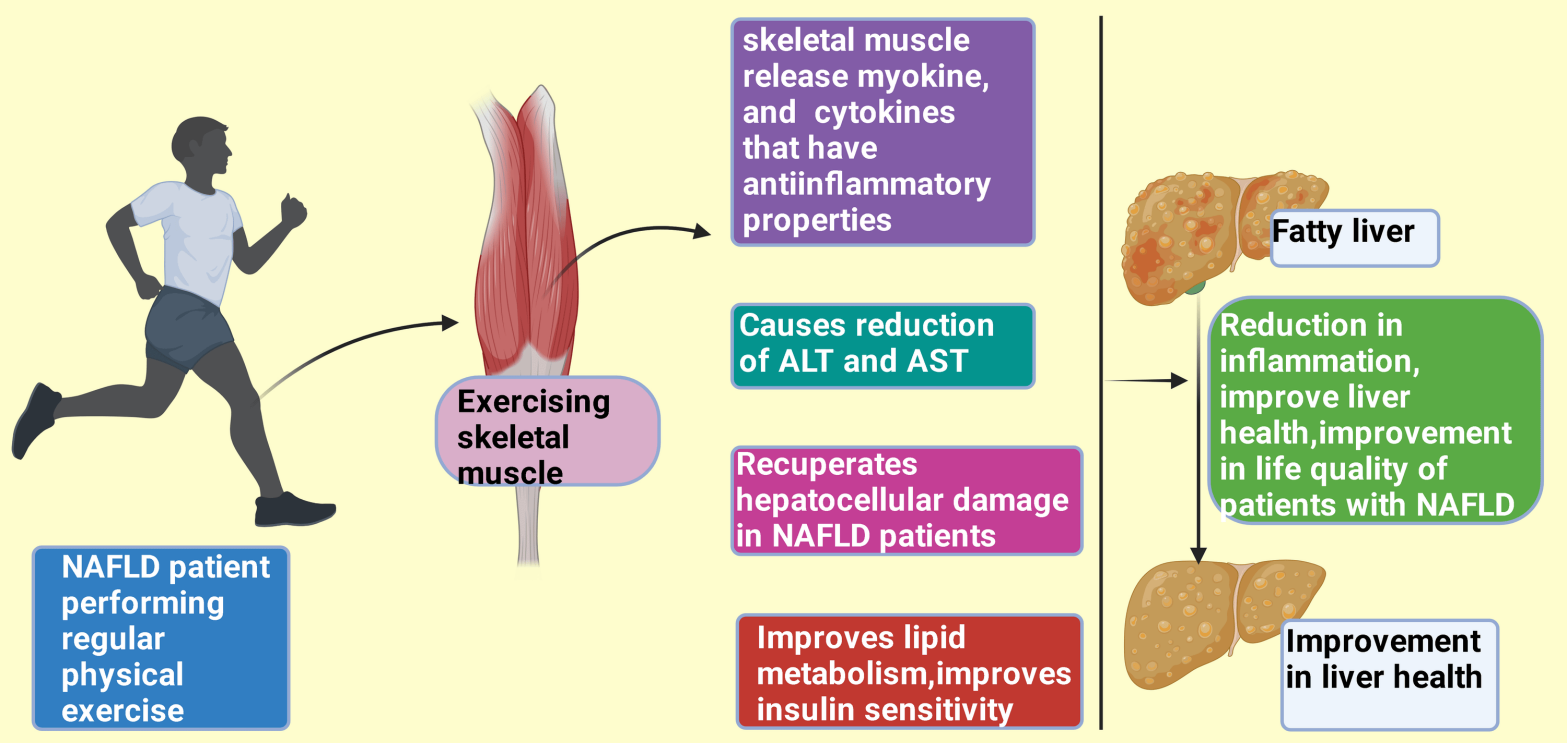
Physical exercise improves liver health in NAFLD patients. NAFLD: Non-alcoholic fatty liver disease; ALT: Alanine aminotransferase; AST: Aspartate aminotransferase. This figure was drawn using the premium version of BioRender (https://biorender.com/), accessed on September 21, 2024, with the agreement license number AB27C1IKPY [10]. Image credit: Rahnuma Ahmad

Furthermore, moderate to intense physical activity considerably reduces alanine aminotransferase (ALT) and aspartate aminotransferase (AST) and recuperates the hepatocellular damage among individuals with NAFLD [114,119]. High-level ALT and AST indicate hepatocellular injury (Figure 4) [120,121]. Xue et al. (2024) conducted one systematic review and meta-analysis and reported that regular physical activity reduces hepatic fat substances, fosters blood lipid metabolism, and improves the quality of life among patients suffering from NAFLD [122].

Pharmacological Intervention for the Management of NAFLD

To date, the United States Food and Drug Administration (USFDA) and European Medicines Agency (EMA) have not approved any medication for the management of NAFLD [123]. Consequently, any medication presently utilized for treating NAFLD must be considered "off-label use" [124,125]. Multiple systematic reviews and meta-analyses reported that medication for T2DM, hyperlipidemia, and other issues of MetS that USFDA and EMA approve for mentioned diseases is often used and improves NAFLD [126-131]. Insulin resistance (IR) is a foremost procedure in the evolution and progression of NAFLD [132-136]. Hence, medications possess potential pharmacodynamics to increase insulin sensitivity, congregating much attention for utilization for NAFLD or non-alcoholic steatohepatitis (NASH) (the most severe form of NAFLD) [137-141]. Kumar et al. reported that sustained high blood glucose levels promote diverse impediments comprising renal disorders, hepatic cirrhosis, and HCC [142]. Myriad aspects cause the development of liver-related disorders, including HCC involving IR and oxidative stress [143,144]. Oxidative stress remains a critical issue in the evolution of IR and DM, as well as many other impediments of DM, such as microvascular and cardiovascular issues [145]. Multiple studies reported that oxidative stress promotes the synthesis of ﻿insulin-degrading enzyme (IDE) and biliverdin reductase-A (BVR-A), thereby causing IRV [146,147].

Thiazolidinediones (TDZs), e.g., pioglitazone, rosiglitazone [148,149], glucagon-like peptide-1 (GLP-1) receptor agonists (GLP-1RAs), e.g., lixisenatide, liraglutide, dulaglutide, semaglutide [148,150,151] and sodium-glucose transport protein 2 (SGLT2) inhibitors, e.g., empagliflozin, dapagliflozin, canagliflozin, and ertugliflozin [152,153] are effective in controlling the blood glucose level, reducing risk of cardiovascular diseases, and giving the positive clinical outcome of diverse liver disorders including NAFLD [154-156]. Medications, TDZs [148], GLP-1RAs [150], and SGLT2 antagonists, control hyperglycemia, lower HbA1C [157], and improve T2DM and cardiovascular issues, favorable alteration of serum lipid profile, and additionally fatty degeneration of the liver. Multiple research projects revealed that diet control with a wholesome, balanced diet and increased physical activity remain predominant features for managing NAFLD [158-160].

Metformin and Non-alcoholic Fatty Liver Disease

**Figure 5:**
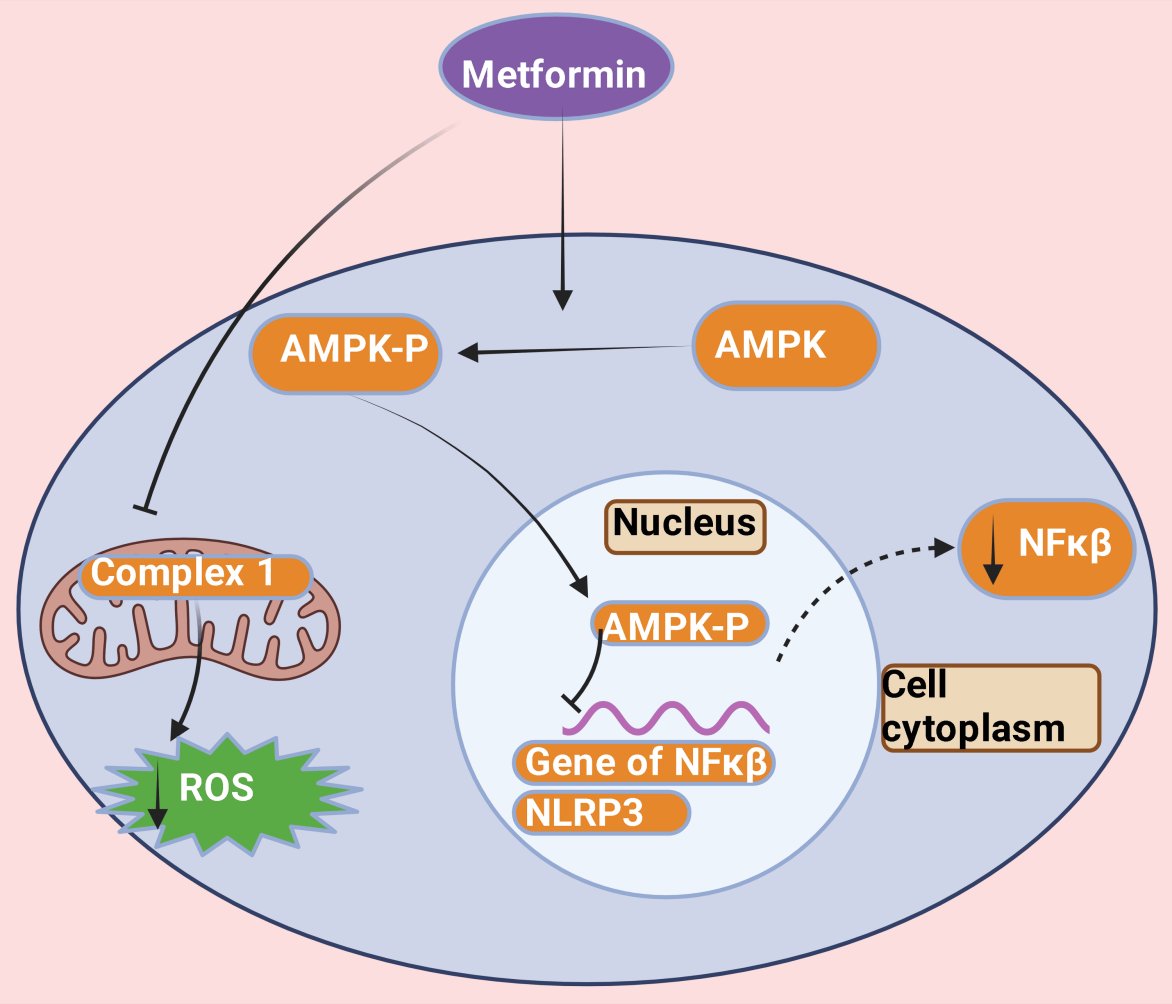
Role of metformin in reducing the production of reactive oxygen species, nuclear factor kappa beta, and NLRP3. Metformin phosphorylates AMPK, which enters the nucleus and impedes NF-κB signaling and nucleotide-binding domain, leucine-rich family, pyrin domain-containing-3 (NLRP3) inflammasome course and restricting reactive oxygen species (ROS) synthesis by inhibiting complex 1 in mitochondria. NF-κB: nuclear factor kappa beta; NLRP3: nucleotide-binding domain, leucine-rich–containing family, pyrin domain–containing-3; ROS: reactive oxygen species; AMPK: AMP-activated protein kinase; AMPK-P: phosphorylated AMP-activated protein kinase. This figure was drawn using the premium version of BioRender (https://biorender.com/), accessed on September 23, 2024, with the agreement license number OL27CBTPV1 [10]. Image credit: Rahnuma Ahmad

﻿Metformin, a typical insulin sensitizer medication, has been extensively prescribed around the globe among T2DM cases [39,161,162]. ﻿By increasing insulin sensitivity, metformin reduces hyperglycemia IR and controls serum glucose level-induced T2DM [162,163]. Metformin impedes nuclear factor kappa B (NF-κB) signaling [164,165] and nucleotide-binding domain, leucine-rich-containing family, pyrin domain-containing-3 (NLRP3) inflammasome course and restricting reactive oxygen species (ROS) synthesis by macrophages through an AMP-activated protein kinase (AMPK) reliant or self-reliant, demeanor (Figure 5) [166-168]. Thereby, metformin deters the transformation of monocytes into macrophages [169,170]. This process escalates ATP cassette transporter type 1 (ABCA-1) endeavor [171], thereby fostering the dissemination of cholesterol from lipid-rich macrophages and enhancing high-density lipoprotein cholesterol (HDL-c) activity [172]. Therefore, it minimizes leukocyte-endothelium communication [173]. Hence, metformin decreases the inflammatory immune response and improves organ recovery induced by T2DM [174,175]. The long-standing second-rate inflammatory process plays a leading role in several non-communicable diseases because of unrelenting raised intensities of circulating pro-inflammatory cytokines throughout the lifetime [176-179]. These diseases included hepatic (severe form of NAFLD [39,180-182], NASH, fibrosis, cirrhosis, hepatocellular carcinoma) and extrahepatic (cardiovascular diseases, T2DM, renal disorders, etc.) [183-187].

A Brief Portrayal of Metformin Action Regarding the Management of NAFLD

**Figure 6:**
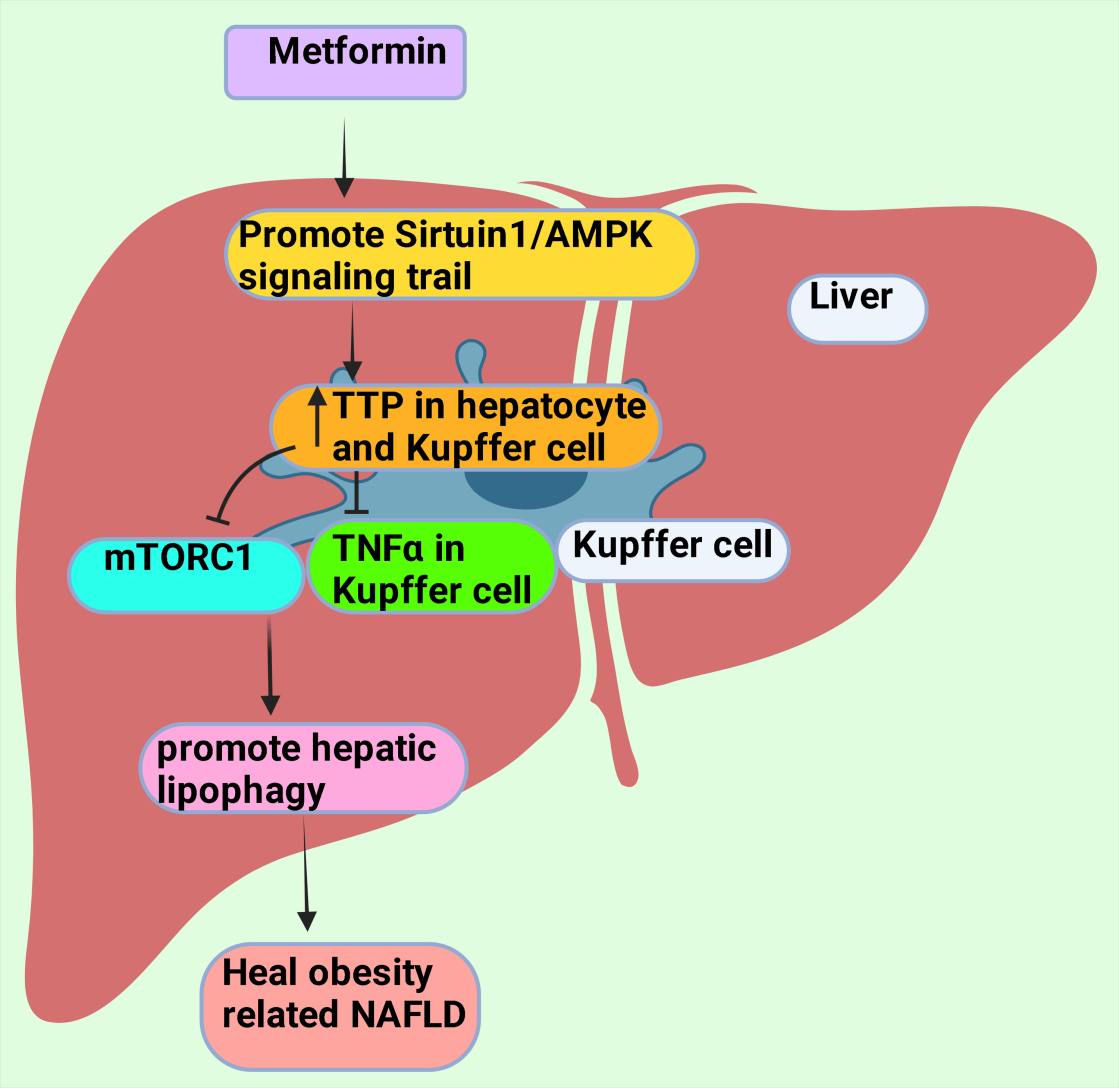
Metformin promotes lipophagy in hepatic cells by inhibiting TNFα and mTORC1 via actuation of TTP through the Sirtuin 1/AMPK signaling trail TNF α: Tumor necrosis factor-alpha; TTP: tristetraprolin; mTORC1: mammalian target of rapamycin complex 1; AMPK: AMP-activated protein kinase. This figure was drawn using the premium version of BioRender (https://biorender.com/), accessed on September 25, 2024, with the agreement license number VX27CL769T [10]. Image credit: Rahnuma Ahmad

Metformin-provoked diminution of severity of NAFLD, possibly because of the amiable roles of Kupffer cells (KCs) and hepatocytes. This process is arbitrated by the existence of an mRNA-binding protein named tristetraprolin (TTP) [188,189]. Metformin actuates TTP in hepatocytes and KCs by the Sirtuin 1 (Sirt1)/AMPK signaling trail [190,191]. TTP inhibits the synthesis of tumor necrosis factor-alpha (TNF-α) in KCs, resulting in a drop in hepatocellular necroptosis [187]. Metformin stimulates TTP activation that deters the mammalian target of rapamycin complex-1 or mechanistic target of rapamycin complex 1 (mTORC1) through undermines Ras homolog enriched in the brain (RHEB) [192,193]. It ultimately upholds transcription factor EB (TFEB) and causes the nuclear transfer to foster hepatic cell lipophagy (a particular type of autophagy), healing obesity-related NAFLD (Figure 6) [188,190,194].

Multiple fatty hepatic genes patatin-like phospholipase domain-containing protein 3 (PNPLA3), transmembrane 6 superfamily member 2 (TM6SF2), hydroxysteroid 17-beta-dehydrogenase 13 (HSD17B13), and membrane-bound O-acyltransferase domain-containing protein 7 ((MBOAT7) also known as lysophospholipid acyltransferase 7) are responsible for the development NAFLD and NASH have been identified [195-197]. Multiple studies reported that the rs738409 variant of the PNPLA3 gene was responsible factor for progression of hepatic fibrosis, NAFLD/NASH, and higher risk of emerging HCC [198-200] among Japanese [201], Hmong population (currently Hmong people principally live in countries in Southeast Asia such as Myanmar, Thailand, Vietnam, Laos, and also in Southwest China) [202], Brazil (both among whites, blacks, and pardo) [203] multi-ethnic group of Malaysia (Malay, Chinese, and Indian) [204], Thailand [205], Guatemala [206], and many other countries. PNPLA3 polymorphism variant rs738409 plays a typical and mightiest gene in developing NAFLD [207,208]. Metformin modifies NAFLD in the development of gene expression [39,209]. These genes are responsible for inflammation in hepatic issues [208], thereby reducing hepatic fibrosis and stiffness and improving NAFLD [209]. It has been reported that metformin 500 mg 3 times daily for four months reduces hepatic transaminase (both ALT and AST) concentrations and improves hepatic insulin sensitivity [210]. Krakoff et al. (2010) reported that metformin steadily lowers serum ALT; nevertheless, body weight management and lifestyle alteration remain the principal priorities of the NAFLD therapeutic intervention strategy [211]. Multiple studies reported that metformin minimizes serum AST and ALT levels, improves hepatic physiology, vital body measurements, homeostatic model assessment for insulin resistance (HOMA-IR), body mass index (BMI), hepatic steatosis index (HSI), and metabolic variable among cases NAFLD with or without distinction DM [212-215]. However, Zhang et al., in their network meta-analysis, reported that metformin had a positive impact in minimizing ALT levels. Nevertheless, saroglitazar (a dual peroxisome proliferator-activated receptor (PPAR) α/γ agonist) efficacy was higher in comparison to metformin [216]. The Drug Controller General of India (DCGI) approved saroglitazar to treat NAFLD and NASH in March 2020, and earlier, it was permitted for diabetic dyslipidemia and hypertriglyceridemia [217-219]. ﻿Nevertheless, saroglitazar has not been allowed for NAFLD and NASH by drug regulatory authorities of several countries around the globe [220]. Saroglitazar, up to the present time, is in phase 2 trial in the USA and has yet to be approved by the FDA for NAFLD and NASH [221]. Huang et al. 2022 propose that metformin could be a repositioning medication for the treatment of NAFLD with high levels of ALT, AST, triglyceride (TG), total cholesterol (TC), and IR [182]. Another metanalysis revealed that TDZs, GLP-1RAs, and metformin (in particular, pioglitazone) were the most promising therapeutic appears for pharmacological therapeutic options for NAFLD management; nevertheless, considerable weight gain remains as ADRs [222]. Similarly, as Petrie (2024) reported in his review paper, metformin, regarding the management of ﻿metabolic dysfunction-associated steatotic liver disease ((MASLD) (previously designated as NAFLD), should be considered a repurposing medicine [223]. Gkiourtzis et al. (2023) in their meta-analysis utilized pediatric NAFLD patients, placebo, and metformin as control and experimental groups, respectively, revealed adequate safety issues or minimum ADRs. Metformin possesses pharmacodynamics in improving insulin and lipid-related parameters among pediatric obese NAFLD cases [224].

A Concise Depiction of Other Serum Glucose-Lowering Agents Except Insulin for the Management of Non-Alcoholic Fatty Liver Disease

It has been reported that worldwide, T2DM and NAFLD are increasingly living together as opposite sides of the same coin among several patients [225-227] because of ﻿the diverse bidirectional nexus [228] and ﻿drastically mortifies prognosis of these cases [225]. Dharmalingam et al. 2018 reported that among T2DM sufferers, 70% concurrently had NAFLD [229]. Scheen (2023) reported that medical doctors were disinclined to prescribe glucose-lowering agents other than insulin among patients with T2DM and fatty liver disease for many decades [230]. This study further reported that novel glucose-lowering medicines, such as GLP-1RAs and SGLT2 antagonists, lever up new horizon aspiration. These medications possess minimum (tolerable) ADRs and trigger weight loss, pleiotropic phenomenon (a distinct gene provides manifold phenotypic attributes), and safeguard cardiorenal physiology, as evidenced in efficacious therapeutic outcomes in managing MAFLD [230]. Jang et al. (2024) in their original research, reported that among multiple (TDZs, ﻿SGLT2 blockers, dipeptidyl peptidase 4 (DPP-4) antagonists, and sulfonylureas) orally prescribed antidiabetic medication, SGLT2 antagonists possibly would somewhat better choice among patients with NAFLD and T2DM. This study advocated more long-term research in this area to decide to shift in prescribing practices [231]. Park et al. (2023) reported that GLP-1RAs possess better pharmacodynamics in minimizing ﻿ BMI, waist circumference, and hepatic fat portion among patients with NAFLD and NASH who are overweight or obese compared to TZDs [232].

Long-Term Adverse Drug Reactions of Metformin

Metformin is primarily considered a drug of choice and is heavily prescribed for T2DM [233,234]. Metformin is otherwise safe and well-tolerated medication if consumed for a prolonged period [235]. The most considerable ADRs of metformin allergies include lactic acidosis, vitamin B_12_ deficiency, metallic (altered) taste, and gastrointestinal disorders (nausea, vomiting, and diarrhea) [236]. Brand et al. (2022) reported that when prescribed among pregnant subjects, metformin singly or metformin + insulin does not produce additional ADRs or risk features compared to insulin [237]. Liu et al. (2024) revealed that prolonged consumption of metformin increased the possibility of ΔFosB degradation [238]. ΔFosB is a Fos close relative of transcription factor proteins [239,240]. Hence, degraded ΔFosB impairs the evolution of levodopa-induced dyskinesia (LID) synthesis by initiating the AMPK-facilitated autophagy route. This study furthermore provides evidence that the AMPK-persuaded autophagy passageway is a unique therapeutic goal for LID and signifies that conceivably repositing metformin is an advantageous therapeutic contestant for LID [238]. Long-term overdose of metformin could upregulate hydrogen sulfide (H_2_S) levels in the liver cells, causing hepatocellular damage in animal models [241,242].

Consequently, constant pharmacovigilance is an urgent necessity to monitor the H2S level in hepatic tissue; thereby, sharp diagnosis and pre can be executed [238]. Nevertheless, Conde et al. reported that metformin has novel protective mechanisms of metformin and indicated that repositioned metformin has the probability to be a novel treatment alternative for the management of oxidative stress-connected hepatic disorders [243]. Patients with renal and hepatic disorders develop lactic acidosis because of severe overdose of metformin and poor elimination [244-246]. These patients frequently and gradually develop symptoms like abdominal pain, nausea, hypotension, tachycardia, and tachypnea [244]. Furthermore, increased levels of lactic acid can lead to severe acidemia, tissue hypoperfusion, hypoxia, cardiopulmonary failure, acute renal damage, and hepatic dysfunction [244,247,248]. Metformin provokes vitamin B_12_ improper absorption from the gastrointestinal tract and raises the possibility of the risk of vitamin B_12_ scarcity among T2DM cases, especially after 12 to months of use [249-252]. Kim et al. (2019) reported taking metformin 1.5 gm daily or more principally related to vitamin B_12_ deficiency. It has been suggested that multivitamin supplementation frequently alleviates vitamin B_12 _insufficiency [253]. 

The Principal Findings of This Narrative Review

This narrative review highlights that metformin, which is typically used to control T2DM, polycystic ovarian syndrome (PCOS), and body weight, is now believed to be of use in improving several other conditions like NAFLD, liver cirrhosis, various carcinomas including liver carcinoma [33-62]. NAFLD is aggravated by inflammation, oxidative stress, and insulin resistance. NAFLD is now a global public health concern, with up to 32% of the worldwide adult population suffering from it [67]. Therapeutic management classically includes weight reduction through altering lifestyle, which includes regular physical exercise and adopting eating habits (nutritious food that excludes energy-dense, carbohydrate-rich) [87-93]. Research also suggests that hypoglycemic agents like Thiazolidinedione and sodium-glucose transporter protein inhibitors may improve liver health by lowering blood glucose levels [148,149]. Metformin shows several mechanisms by which it may promote liver health in conditions like NAFLD. It improves insulin sensitivity and inhibits nuclear factor kappa beta, NLRP3, and ROS. Conversion of monocyte to macrophage is deterred by metformin, and cholesterol is disseminated from lipid-rich macrophage with an increase in high-density lipoprotein (HDL). Metformin also promotes TTP that mTOR, TNFα and accelerates lipophagy, healing obesity-associated NAFLD [162-172, 188,190,194]. Metformin has been noted to suppress the expression of genes that aggravate fatty liver [39,209]. Pharmacovigilance is required while using metformin for a long time since there may be a formation of hydrogen sulfide, which may cause liver damage [238,241,242]. Other adverse reactions include gastrointestinal disorders, metallic taste in the mouth, lactic acidosis, and vitamin B12 deficiency [236]. 

Limitations of This Study

Narrative reviews have inbuilt constraints regarding neutrality, comprehensiveness of literature exploration, and clarification of results [254]. Nonetheless, Greenhalgh et al. (2018) reported that narrative reviews deliver clarification and appraisal, and strategic input snowballs conception and comprehension [255].

Future Research Perspectives

However, multiple studies have reported that metformin is a possible therapeutic contestant for the pharmacological intervention of NAFLD [39,182,224]. Nonetheless, these papers [39,182,224] recommend future research to get approval from several necessary drug regulatory authorities and national and international guidelines [88,126,256-258].

## Conclusions

Although metformin is known for its role in managing T2DM, PCOS, and weight reduction, studies have emerged indicating its healing effect in cases of diseases like NAFLD, liver cirrhosis, and several carcinomas. NAFLD comprises a considerable portion of the world's adult population, and a much higher proportion exists among the pediatric obese population. The first steps in the management of NAFLD include adopting a healthy lifestyle like eating a nutritious diet, avoiding an energy-dense carbohydrate-rich diet, lowering food portions, and regular physical exercise to reduce weight and several hypoglycemic agents like thiazolidinedione and sodium-glucose cotransporter-2 blocker can be part of the management. Metformin plays several roles in improving liver health by inducing lipophagy and reducing oxidative stress and inflammation. It may even reduce gene expression that promotes fatty liver, hepatic fibrosis, and stiffness, like PNPLA3 polymorphism variant rs738409. Thus, metformin may reduce hepatic fibrosis and stiffness by reducing inflammation. There may be some complications due to long-term consumption of metformin that include lactic acidosis, vitamin B12 deficiency, metallic taste in the mouth, and gastrointestinal disorders like abdominal pain, nausea, and vomiting. Pharmacovigilance is required since a long-term overdose of metformin may raise hepatic cell hydrogen sulfide levels. Further research should be carried out to understand the protective mechanisms of metformin and the appropriate dosage for different liver diseases. It should be considered while forming national and international guidelines for managing NAFLD.
